# How to avoid one thousand opportunities to do harm in genomic medicine

**DOI:** 10.1186/1753-6561-6-S6-O13

**Published:** 2012-10-01

**Authors:** Isaac Kohane

**Affiliations:** 1 Children's Hospital Boston, MA, USA

## 

With the advent of whole genome sequencing made clinically available, the number of incidental findings is likely to rise. False positive incidental findings are of particular clinical concern, and they can usefully be classified into four categories. In order of increasing challenge, there is first, the substantial proportion of 'textbook cases' of mutations documented to cause human disease in a highly penetrant Mendelian fashion, which are incorrectly annotated in the databases. The second is the technical/measurement error rate in genome-scale sequencing. Third is the incorrect assignment of prior probabilities for much of our genetic and genomic knowledge. The fourth derives from testing multiple hypotheses across millions of variants. I will describe the nature of these components, provide rough estimates for the magnitude of the problem and point out existing approaches that will serve to control the growth of these aspects of the incidentalome.

